# A novel 3D printed mechanical actuator using centrifugal force for magnetic resonance elastography: Initial results in an anthropomorphic prostate phantom

**DOI:** 10.1371/journal.pone.0205442

**Published:** 2018-10-08

**Authors:** Wiebke Neumann, Andreas Bichert, Jonas Fleischhauer, Antonia Stern, Roxana Figuli, Manfred Wilhelm, Lothar R. Schad, Frank G. Zöllner

**Affiliations:** 1 Department of Computer Assisted Clinical Medicine, Medical Faculty Mannheim, Heidelberg University, Mannheim, Germany; 2 Institute for Chemical Technology and Polymer Chemistry of Karlsruhe Institute of Technology, Karlsruhe, Germany; University of Montreal, CANADA

## Abstract

This work demonstrates a new method for the generation of mechanical shear wave during magnetic resonance elastography (MRE) that creates greater forces at higher vibrational frequencies as opposed to conventionally used pneumatic transducers. We developed an MR-compatible pneumatic turbine with an eccentric mass that creates a sinusoidal centrifugal force. The turbine was assessed with respect to its technical parameters and evaluated for MRE on a custom-made anthropomorphic prostate phantom. The silicone-based tissue-mimicking materials of the phantom were selected with regard to their complex shear moduli examined by rheometric testing. The tissue-mimicking materials closely matched human soft tissue elasticity values with a complex shear modulus ranging from 3.21 kPa to 7.29 kPa. We acquired MRE images on this phantom at 3 T with actuation frequencies of 50, 60 Hz, 70 Hz, and 80 Hz. The turbine generated vibrational wave amplitudes sufficiently large to entirely penetrate the phantoms during the feasibility study. Increased wave length in the stiffer inclusions compared to softer background material were detected. Our initial results suggest that silicone-based phantoms are useful for the evaluation of elasticities during MRE. Furthermore, our turbine seems suitable for the mechanical assessment of soft tissue during MRE.

## Introduction

Magnetic resonance elastography (MRE) is a non-invasive imaging technique used for the quantification of spatial stiffness of soft tissues during MR examinations [1–8]. It can serve as an estimator of the mechanical properties of living tissue and may be used as a discriminator for benign or cancerous tissue [9].

During an MRE examination, a dynamic harmonic mechanical excitation is applied to the body, generating shear waves, which propagate through the tissue. The mechanical response of the tissue can be measured using phase-contrast MRE sequences synchronized to the applied vibrations [10]. These acquired displacement fields are then mathematically converted into spatial stiffness maps [11,12].

In general, the quantification of tissue stiffness can be performed most efficiently in organs close to the body surface, due to the propagation characteristics of mechanical shear waves. In the case of isotropic, homogeneous materials such as the liver, an estimation of the elasticity modulus can easily be derived from the wave speed [13]. Hence, MRE is widely used in the assessment of liver fibrosis and it may replace tissue biopsy [14].

One of the main obstacles remains a reliable wave induction for other clinical applications such as the prostate, pancreas and kidney, as well as heart and lung for MRE [15–19]. Overall, high actuation frequencies (> 150 Hz) are beneficial to resolve small lesions. However, wave attenuation in soft tissue is also stronger at higher actuation frequencies, a dilemma particularly in deep-lying tissues. Compared to lower excitation frequencies, the problem of wave attenuation leads to a reduced signal to noise ratio [20].

Several methods currently exist for dynamic harmonic mechanical excitation. The most commonly used systems in earlier MRE studies are acoustic driving systems. Here, shear waves are generated using pneumatic cushions powered by varying acoustic pressure levels. These sinusoidal sound waves are generated by an active audio device located outside the scanner room [21,22,23] at frequencies in the range of 40 Hz to 200 Hz [24]. The pneumatic drum drivers operate well in the lower frequency regime (≈ 60 Hz). At higher frequencies, however, sustaining sufficiently large wave amplitudes becomes problematic and additional power amplification is necessary to maintain an adequately large displacement range [18,25].

Other application-specific drivers have been proposed that use electromechanical coils [26] or piezoelectric drivers [27], although these electromechanical actuators can generate image artifacts, create a heat build-up typical for electromechanical drivers [21], or need to be actively shielded [28].

A further transducer concept, the gravitational transducer, is also driven by a rotational eccentric mass based on a similar approach as the herein presented driver [29]. Yet, it is powered by a stepper motor that is attached to the gravitational transducer via a rotating rod making the set-up in the scanner room cumbersome and limiting the accessible surfaces.

This study set out to investigate the feasibility of a novel method for sinusoidal mechanical wave generation based on the principle of centrifugal force. Our design is similar to that of the industrially used compressed air vibrators, which are common in the bulk material handling sector. There, an eccentric weight within these pneumatic turbines, also called unbalance, generates a dynamic harmonic vibration. This results in a centrifugal force with its amplitude depending on the driving frequency of the turbine as well as on the weight and dimensions of the unbalance. The frequency itself can be freely selected according to the applied air pressure level. However, due to the turbine material and centrifugal force range, commercial compressed air vibrators cannot be operated safely within high magnetic fields (> 1 T) nor are in the required range of mechanical wave actuation force.

We have developed a new 3D printed pneumatic vibrator that is MR-safe and corresponds to the range of wave amplitudes needed to generate suitable shear waves in human tissue for MRE. One purpose of this study was to assess the technical parameters of the actuator. Furthermore, it describes the manufacturing process of tissue-elasticity-mimicking phantoms. Finally, this paper presents results an MRE study on our in-house developed phantom at 3 T with the novel actuator. A preliminary version of this work has been reported before [30] describing the fundamental design of the pneumatic vibrator for generating shear waves.

## Methods

The first two sections discuss the design and implementation of the actuator divided into the passive pneumatic turbine located in the scanner room and the active driver controlling the pressure of the compressed air located in the control room. The other sections describe the methodology of evaluating technical parameters and phantom studies on tissue-mimicking material. The generation of shear waves with a frequency of 60 Hz is the most widely used actuation frequency in clinical applications at present [31,32] and the reason this work focused on this particular actuation frequency of 60 Hz.

### Design of pneumatic turbine

**Fig 1:**
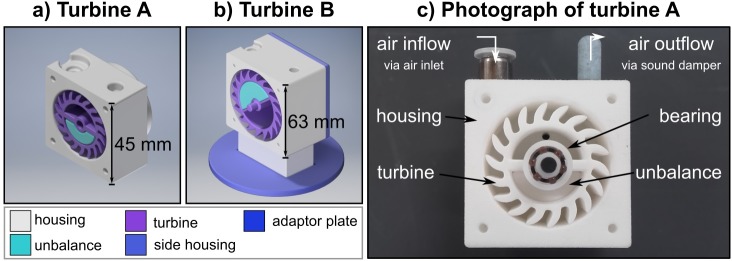
The components of the pneumatic turbines. a) CAD schematic of turbine A. b) CAD schematic of turbine B. c) Photograph of turbine A. Compressed air is supplied to the turbine via the air inlet (top left). The inserted unbalance causes a dynamic harmonic excitation. The compressed air exits the turbine through the sound damper (top right). The frontal side housing of the pneumatic turbine is removed for a clearer representation.

The passive part of the actuator consisted of a compressed air turbine that was placed on the volume under investigation (Fig 1). The turbine was 3D printed and created a centrifugal force during rotation due to an unbalance within the turbine.

In general, the generated force F depends on the weight of the unbalance m_ecc_, the distance r_ecc_ between the mass center of gravity of the unbalance and the rotation center of the turbine, as well as the angular velocity *ω* of the turbine and can be calculated via *F* = *m*_*ecc*_*r*_*ecc*_*ω*^2^.

Consequently, a greater force can be generated by: (1) use of materials with higher densities for the unbalance, (2) change in geometry so that the distance of the center of gravity of the unbalances increases to the center of rotation, (3) increase in the volume of the unbalance, or (4) increase in the frequency of the turbine.

Two turbines were designed and built for this study. Turbine A is distinguishable to turbine B mainly due to a smaller overall geometry, higher maximum actuation frequency and varying weight of the unbalances.

Magnetic components cannot be used because the turbine is located within the scanner room and therefore exposed to high magnetic fields (up to 3 T in our study). Hence, we chose polyamide (PA 12) for all 3D printed parts. The material had a tensile strength of 48MPa ± 3 MPa (according to DIN EN ISO 527) and a heat deflection temperature of 86°C (according to ASTM D648 (1.82 MPa)). The components were designed with CAD software and produced by selective laser sintering. The rolling-element bearings were made of the thermoplastic polyoxymethylene and glass (according to DIN 625–626). The sound damper was composed of plastic. The valve, which served as the inlet for compressed air into the turbine, was the only metallic component. However, it was made of non-magnetic brass and was not subject to any forces during MR measurements.

### Design of active driver

**Fig 2:**
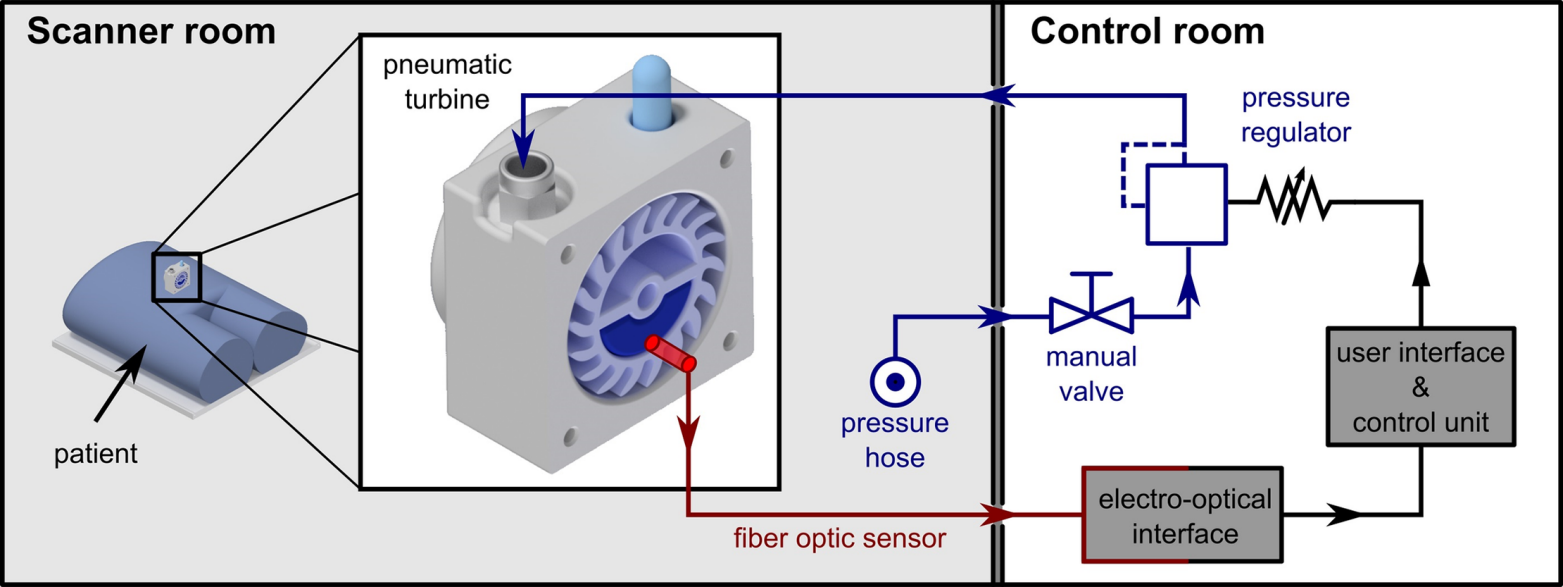
The configuration of the actuator and control unit. Compressed air is supplied via a pressure hose, which is available in all scanner rooms in the clinic (left). All magnetic and active electronic parts are located in the control room (right) and comprise the active driver system. The compressed air is fed to the proportional pressure regulator. The output pressure is regulated by a control voltage. During start-up, the control voltage, i.e. the output pressure, is increased until the nominal frequency of the pneumatic turbine is reached. A fiber optic probe attached to the housing of the turbine provides feedback over the current frequency.

All MR-unsafe and active electronic components were located outside the scanner room and comprised the active part of the actuator (Fig 2). The active driver regulated the pressure of the compressed air that drove the turbine to achieve a certain rotational frequency. A proportional pressure regulator was connected to the in-house pressure hose. The pressure hose is installed in all scanner rooms in the clinic (DIN 13260–2 compliant) and supplies compressed air with a nominal pressure of p_hose_ = 5 bar. The proportional pressure regulator set the output air pressure by adjusting the control voltage in a range from 0 V to 10 V corresponding to the minimal and maximum pressure output.

The probe of an MR-safe fiber optic sensor was mounted on the side housing of the turbine and provided feedback on the rotational frequency of the turbine. During one full rotation of the turbine, the fiber optic sensor detected two signals (low and high). An 8-bit microcontroller (PIC16F1719, Microchip Technology Inc., Germany) on a development board (Explorer 8, Microchip Technology Inc., Germany) evaluated these signals for a time interval of one second and updated the rotational frequency of the turbine. The determined rotational frequency fed into a control loop that regulated the control voltage, i.e. the output pressure, of the proportional pressure regulator. Thus, the output pressure of the compressed air was increased in order to obtain a higher rotational frequency and, vice versa, decreased for a lower rotational frequency. During our work, the maximum output voltage was limited to 4.6 V due to hardware restrictions of the development board resulting in the maximum output pressure of p_max_ ≈ 2.1 bar.

In order to ensure controlled compressed air output, various safety features were implemented. Firstly, the pressure regulator was designed to be normally closed, i.e. if no control voltage was applied to the system or in the event of a power failure, the valve of the pressure regulator closed and no compressed air was fed into the turbine. Secondly, the control voltage, i.e. the output pressure, increased gradually (V_step_ = 45 mV corresponding to p_step_ ≈ 20.25 mbar) during start-up, so that a constant communication with the patient could be maintained to ensure that the induced vibration level is acceptable to the patient. Thirdly, an emergency stop button was implemented at the user interface of the active driver, which instantly set the control voltage to 0 V and stopped the outflow of compressed air. Finally, a manual shut-off valve was installed between the in-house pressure hose and the pressure regulator. This allowed the operator to manually stop the compressed air entry into the driver’s system.

### Technical evaluation of mechanical actuator

For each turbine, three unbalances with different weights (m_A1_ = 1.2 g, m_A2_ = 2.3 g, and m_A3_ = 4.5 g (turbine A) and m_B1_ = 3.0 g, m_B2_ = 4.3 g, and m_B3_ = 8.6 g (turbine B)) were constructed from polyamide to investigate their influence on the generated acceleration. The unbalances were 3D printed, had a semi-cylindrical shape and could be interchangeably inserted into the turbine.

Following Runge et al. [29], we recorded the vibration frequency response spectrum of one turbine at frequencies ranging from 20 Hz to 90 Hz with a step width of 10 Hz using a digital accelerometer. The acceleration sensor was attached to the side housing of the turbine. The pneumatic turbine itself was placed on a silicone gel-based phantom during measurement.

Furthermore, the acceleration at frequencies ranging from 30 Hz to 80 Hz with a step width of 10 Hz for both turbine A and turbine B with all unbalances were measured using a digital accelerometer (ADXL345, Analog Devices, MA, USA) with a 13-bit measurement at up to ±14.95 m/s^2^. The acceleration sensor was attached to the side housing of the turbine and the peak linear acceleration of the housing was recorded. The pneumatic turbine itself was placed on a silicone gel-based phantom during measurement.

### Phantom study

MRE was performed on a custom-made tissue-mimicking prostate phantom as a proof-of-principle. Prior studies have evaluated silicone compositions with a physiologically realistic storage modulus, e.g. for breast phantoms [33], a heart-simulating phantom [34], and evaluation of normal and cancerous prostate tissue [35]. Based on those studies and our experiences in design and manufacturing of anthropomorphic phantoms [36], we chose silicone as the main tissue-mimicking material. We examined 11 silicone samples (Table 1) on their viscoelastic parameters prior to the fabrication of the tissue-imitating phantom. Silicone rubbers with a Shore hardness of 0 ShA and 13 ShA were selected as the base material due to its suitable properties in terms of a simple and reproducible manufacturing process and its long-term stability. To adjust elasticity parameters, different concentrations of silicone oil were added. The silicone base and oil were mixed at room temperature, then the catalyst was added and the sample was degassed in a vacuum chamber.

**Table 1:** Complex shear modulus of tested silicone samples.

	Concentration			Shear modulus at 60 Hz (kPa)		
Sample number	SF 13	SF 00	Silicone oil	G*	G’	G”
1	1.00	0.00	0.00	209.50	207.64	27.84
2	0.50	0.50	0.00	108.25	107.05	16.09
3	0.00	1.00	0.00	25.95	25.02	6.89
4	0.00	0.63	0.38	8.64	8.34	2.26
5*	**0.00**	**0.59**	**0.41**	**7.29**	**7.02**	**1.97**
6	0.00	0.56	0.44	6.20	5.91	1.87
7**	**0.00**	**0.53**	**0.47**	**5.17**	**4.93**	**1.56**
8	0.00	0.48	0.52	3.95	3.75	1.24
9***	**0.00**	**0.43**	**0.57**	**3.21**	**3.06**	**0.98**
10	0.00	0.40	0.60	2.72	2.65	0.60
11	0.00	0.31	0.69	1.47	1.47	0.11

*chosen as inclusion A (Bladder)

**chosen as inclusion B (Prostate)

***chosen as background material of the phantom

The complex shear modulus G* was tested using a strain controlled rheometer with a linear frequency sweep ranging from 30 Hz to 100 Hz (step width 2 Hz) and 25 mm parallel plates of Invar (a nickel-iron alloy). Further parameters were: pre-strain of 0.5%, axial force of F_a_ = 1 N, sample diameter d = 25 mm, sample height h = 3 mm and a fixed temperature of T = 26°C. In order to assess the standard deviation *σ* regarding test-retest deviations, sample # 7 was tested five times. For this purpose, the sample was placed in the rheometer, measured and removed from the test set up repeatedly.

The elasticity parameters of the custom-made prostate phantom were matched to literature elasticity values of human bladder [37] and prostate [38–41]. Following the results of G* of the tested samples (Table 1), the concentrations of sample # 5, # 7, and # 9 corresponding to 41%, 47%, and 57% silicone oil were chosen for the manufacturing of inclusions A (bladder) and B (prostate) and the background material, respectively, of the tissue-mimicking phantom. Three small spheres (d = 10 mm with concentrations of 13%, 20%, and 41% silicone oil) were embedded in inclusion B, resembling possible pathologies due to their increased stiffness.

The phantom was then placed in a 3 T MR scanner (Magnetom Trio, Siemens Healthineers, Germany). MRE was performed at actuation frequencies of 50 Hz, 60 Hz, 70 Hz, and 80 Hz employing a motion-encoding spin-echo echo-planar-imaging (SE-EPI) based sequence with an echo time (TE) and repetition time (TR) of TE/TR = 88/3000 ms, a field of view (FoV) of FoV = 200 mm × 200 mm, an acquisition matrix of 96 × 96, and a slice thickness of 5 mm. An elastogram was obtained at a frequency of 80 Hz using the software MRE/Wave (Version 10.01.07, Rochester, MN, USA).

## Results

### Technical evaluation of mechanical actuator

**Fig 3:**
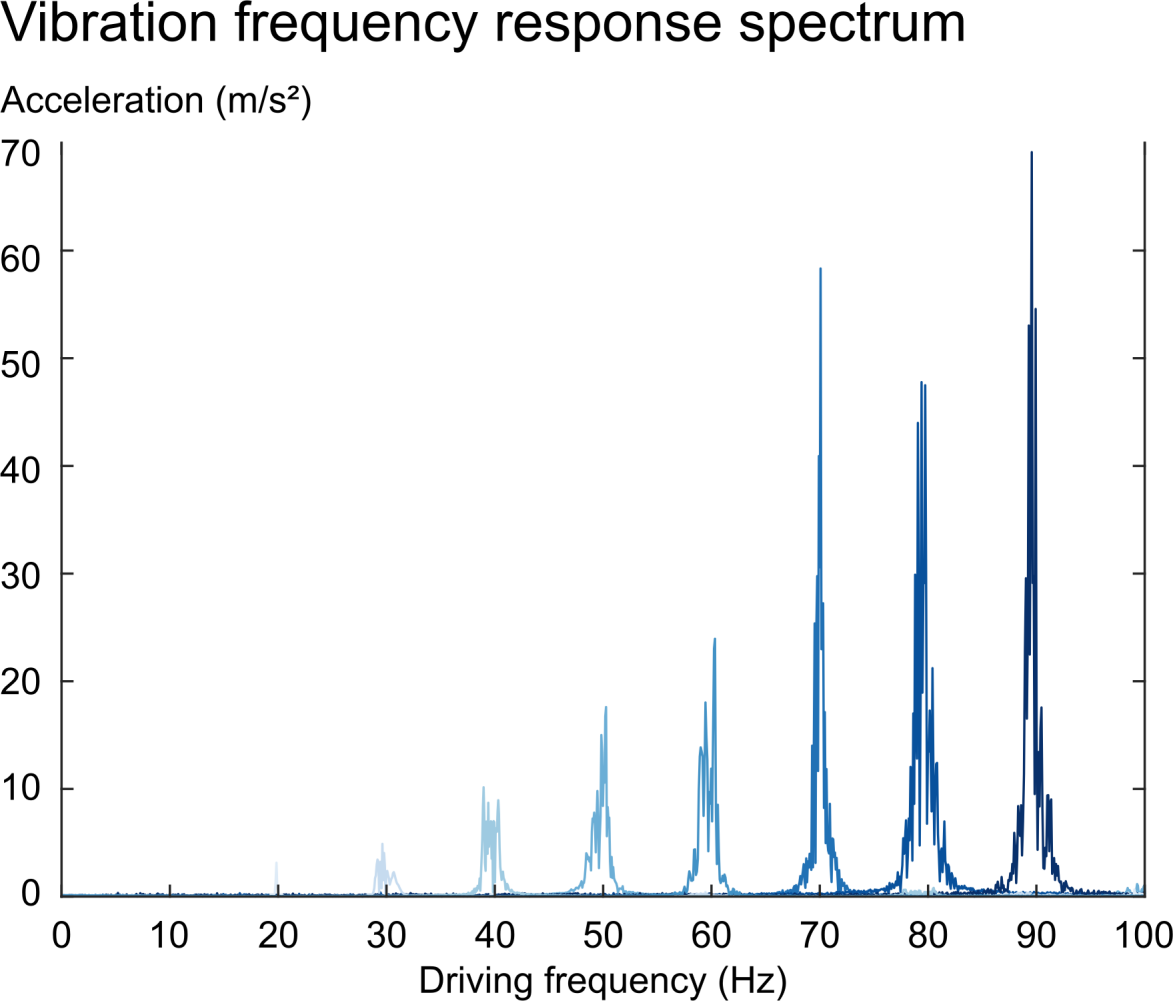
The vibration frequency response of the pneumatic vibrator housing. The vibration frequency response spectrum (in m/s^2^) for frequencies ranging from 20 Hz to 90 Hz with a step width of 10 Hz were evaluated. The acceleration increased with increasing frequency.

The vibration frequency response spectrum increased with increasing frequency (Fig 3) as predicted for such system. An outlier was observed at 70 Hz. Here, the maximum acceleration was larger than it was at 80 Hz. No upper harmonics were present.

**Fig 4:**
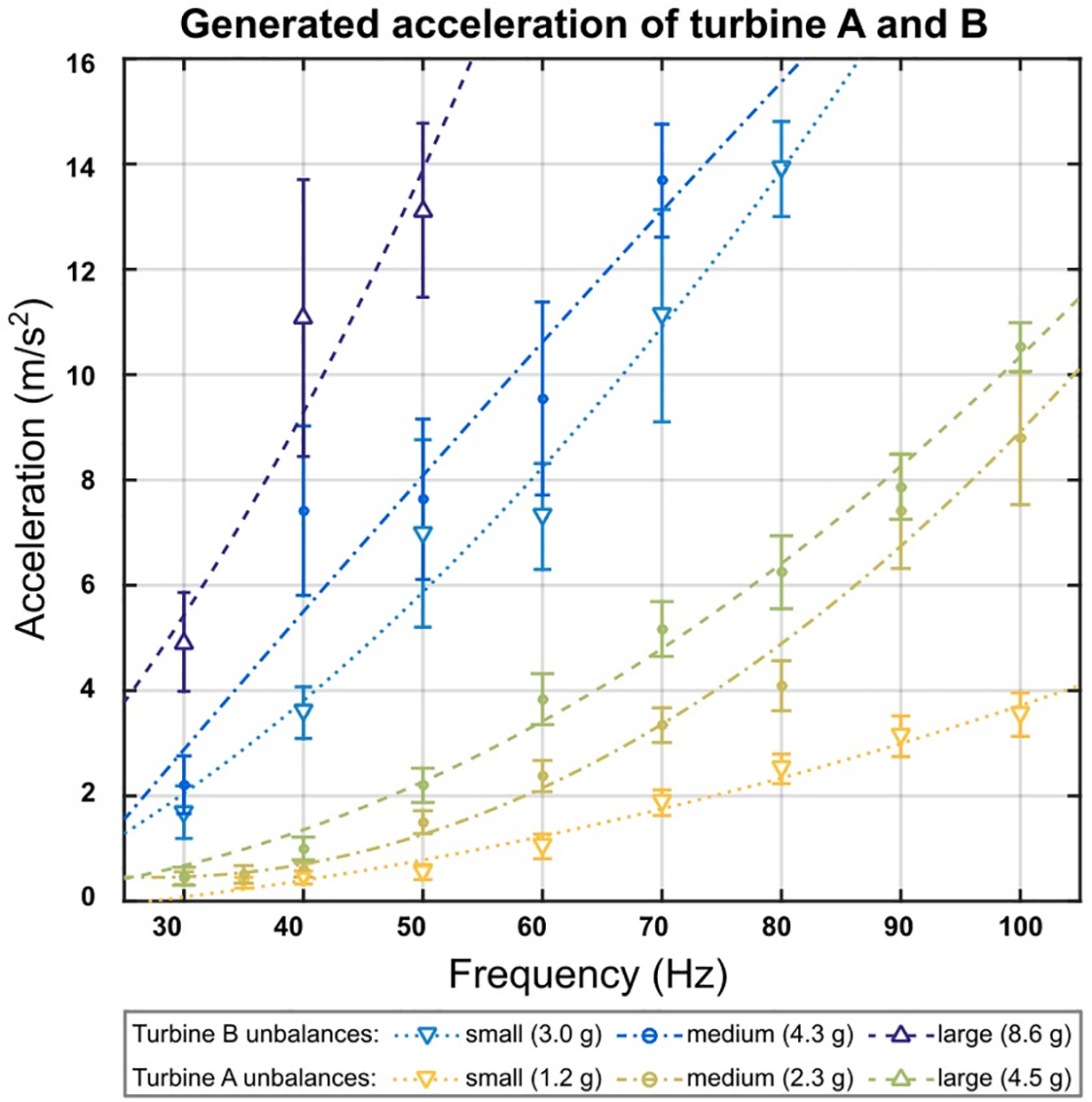
Measured uniaxial acceleration of the pneumatic vibrator acquired by the accelerometer. Unbalances with a weight of m_A1_ = 1.2 g, m_A2_ = 2.3 g, and m_A3_ = 4.5 g (turbine A) and m_B1_ = 3.0 g, m_B2_ = 4.3 g, and m_B2_ = 8.6 g (turbine B) at frequencies ranging from 30 Hz to 100 Hz were evaluated. The maximum measurable acceleration with our current set up was limited to a_max_ < 14.95 m/s^2^ due to the range of the accelerometer and thus no greater values could be recorded.

Acceleration values were a_A1_ = 1.04 ± 0.23 m/s^2^, a_A2_ = 2.38 ± 0.30 m/s^2^, and a_A3_ = 3.84 ± 0.48 m/s^2^ for turbine A and a_B1_ = 7.31 ± 1.01m/s^2^, a_B2_ = 9.55 ± 1.83m/s^2^, and a_B3_ > 14.95 m/s^2^ for turbine B at an actuation frequency of 60 Hz during this feasibility study (Fig 4). The maximum measurable acceleration with our current set up was limited to a_max_ < 14.95 m/s^2^ due to the range of the accelerometer and thus no greater values were recorded. The temporal stability of the system, i.e. frequency shifts over an MRE experiement, was σ_A1_ = 1.0 Hz, σ_A2_ = 1.8 Hz, and σ_A3_ = 1.8 Hz for turbine A and σ_B1_ = 2.9 Hz, σ_B2_ = 2.6 Hz, and σ_B3_ = 0.5 Hz for turbine B at 60 Hz and in the same range for all other tested frequencies.

We were able to infinitively variable the actuation frequencies between 20 Hz to 100 Hz and 30 Hz to 180 Hz for the presented turbines A and B during our experiments. A lower minimum actuation frequency was not possible, as a certain minimum air pressure needed to be maintained to overcome the turbine inertia. The maximum applicable frequency is only theoretically limited by the available in-house air pressure of p_hose_ = 5 bar. During our evaluation, we did not exceed an air pressure level of p_input_ = 2.1 bar but were still well within the range of currently applied wave actuation frequencies for MRE imaging.

### Phantom study

**Fig 5:**
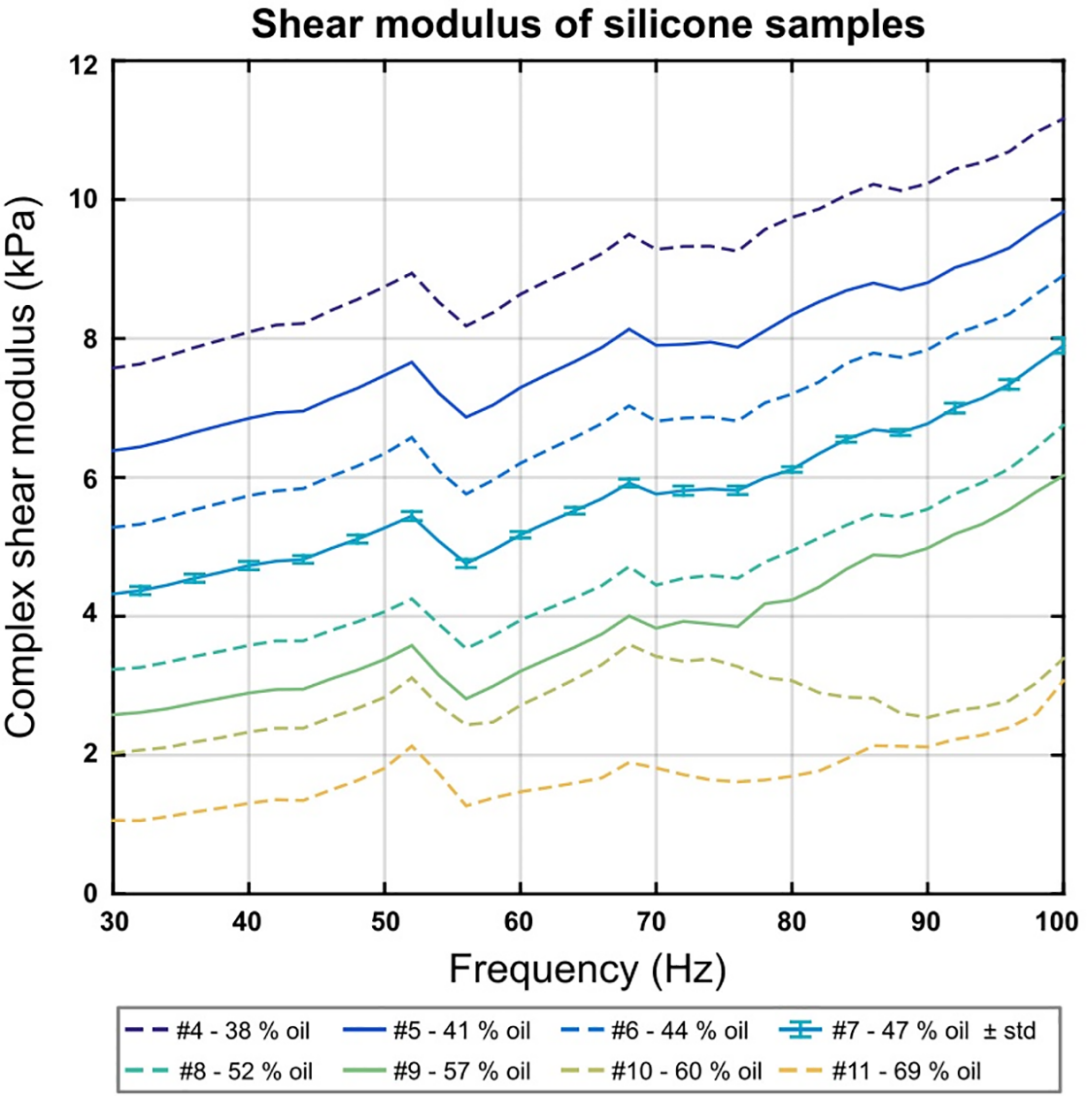
Complex shear modulus G* of selected silicone samples # 4 - # 11 at frequencies ranging from 30 Hz to 100 Hz measured with a strain controlled rheometer. Samples # 1 - # 3 are not shown in this graph as their elasticity is more than three times higher than that of sample # 4. The standard deviation calculated from five re-tests of sample # 7 is also shown. Samples # 5, # 7 and # 9 (**—**) were chosen for the tissue-mimicking phantom, other samples are displayed as—**- -**.

The first set of analyses examined the complex shear modulus G* of the silicone samples at frequencies ranging from 30 Hz to 100 Hz with the strain controlled rheometer (Fig 5). The determined shear moduli of the tested samples were in the range of 1.47 kPa (SF00 silicone rubber diluted with 69% silicone oil) to 209.50 kPa (SF13 silicone rubber without silicone oil) at a frequency of 60 Hz (Table 1). The standard deviation of sample # 7 was determined to be *σ* = 0.06 kPa. The phantom was built using silicone rubber SF00 with 41%, 47%, and 57% silicone oil, corresponding to G* of 7.29 kPa, 5.17 kPa, and 3.21 kPa at 60 Hz.

**Fig 6:**
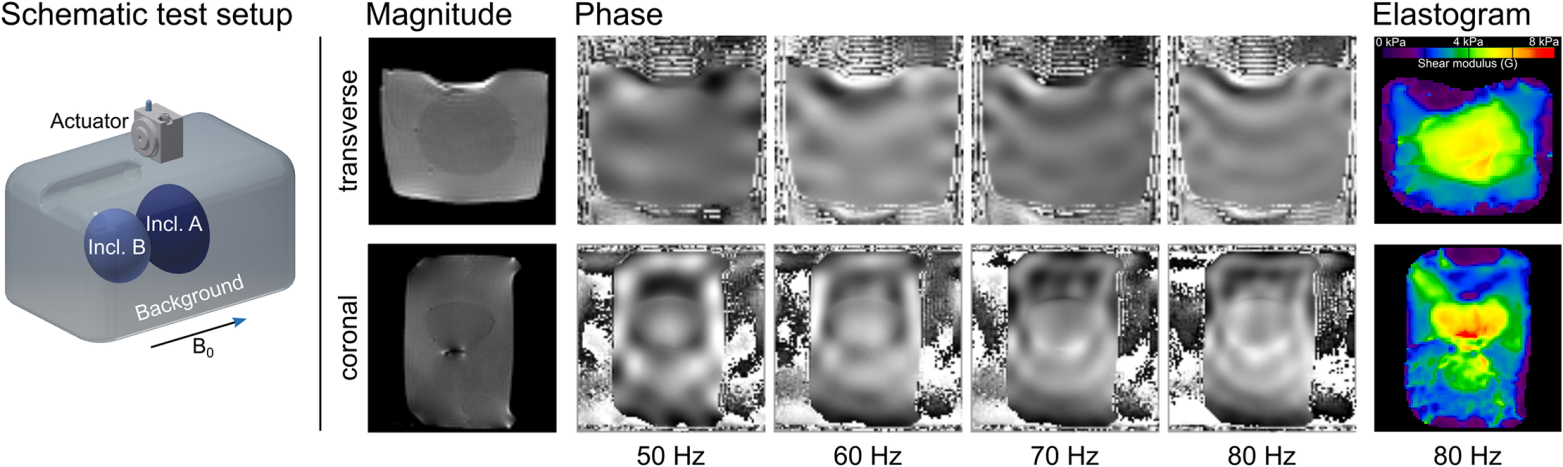
Results of 3 T MRE measurements. Left: Schematic drawing of the phantom used for image acquisition at 3 T. Inclusion A, corresponding to the elasticity of a bladder, is shown in dark blue. Inclusion B, corresponding to the elasticity of a prostate, is depicted in light blue. The actuator was placed on top of the phantom. Middle: Magnitude and phase images. Some trapped air is visible between the inclusions and the background material yielding to artifacts. Phase images were obtained for frequencies ranging between 50 Hz and 80 Hz. Right: Elastograms reconstructed at 80 Hz. Top row is a transverse slice showing inclusion A, Bottom row displays a coronal slice with both inclusions.

We obtained MRE magnitude and phase images at actuation frequencies of 50 Hz, 60 Hz, 70 Hz, and 80 Hz at 3 T. The elastograms obtained for a transverse and coronal slice at 80 Hz showed an increased shear modulus within the inclusions compared to the background material (Fig 6).

**Fig 7:**
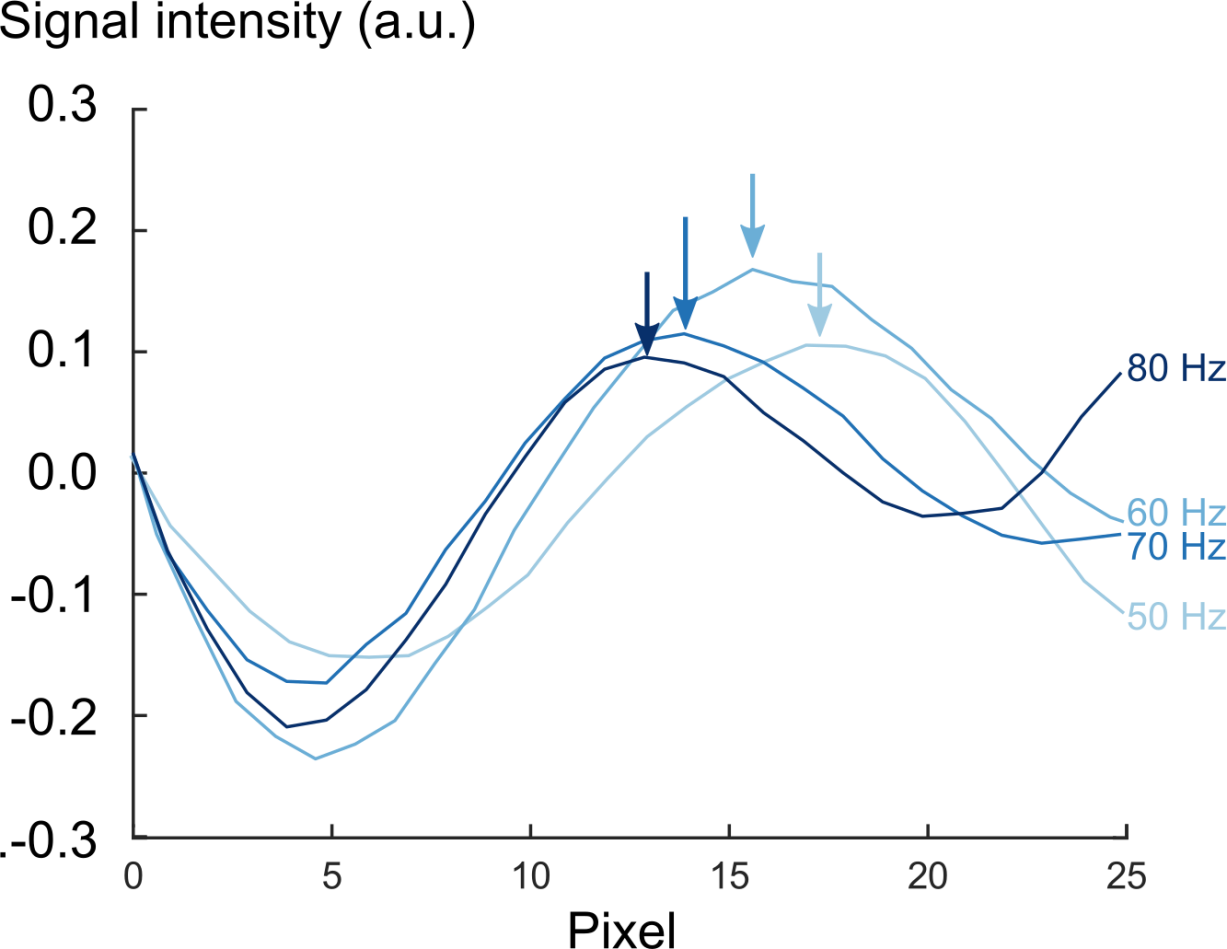
Line profiles of phase signal at 50 Hz, 60 Hz, 70 Hz, and 80 Hz in Inclusion A. A 25-pixel-line was placed in inclusion A and compared at four actuation frequencies. The wave length shortened with increasing frequencies as indicated by arrows at the first maximum of each line profile.

Shear waves generated by our proposed pneumatic turbine propagated through the entire volume of the phantom. The passive driver did not generate any significant artifacts in the acquired MR images. The inclusions A and B, being 2.3 and 1.6 times stiffer than the background material, were clearly visually detectable at 3 T (Fig 7). The manually measured wave lengths in inclusion A was 1.5 times longer compared to the wave lengths in the background material.

## Discussion

The main goal of the current study was to present a new method for controlled dynamic harmonic wave actuation using centrifugal force for the quantification of mechanical properties of soft tissues by MRE.

Our mechanical actuator is an alternative to the conventionally used pneumatic cushions. By using centrifugal forces instead of sound pressure levels, the pneumatic vibrator offers an elegant solution for sufficiently large wave actuation at higher frequencies compared to air cushions where the amplitude of sound pressure waves decreases with increasing frequencies.

In comparison to our presented design, the gravitational transducer by [29] needs a stepper motor connected by a rotating rod. The electric stepper motor needs to be at a certain distance to the scanner. Thus the distance between the stepper motor and transducer yields an increased length of the rod. As the rod transmits the rotation, it needs to be mechanically stable and its rigidity limits the accessible surfaces. Compared to the gravitation transducer, our design omits the need for a rotating rod, as the source of rotation is also the source of vibration. Thus, more surfaces are accessible with our transducer.

A major restriction of MR Elastography for a broader clinical impact is the additionally required software and hardware. The simplicity of our driver configuration also allows other clinics to implement this technology easily. We have demonstrated how the pneumatic vibrator can readily be installed using the in-house compressed air system, 3D printing and a programmable microcontroller unit.

The experimentally determined maximum acceleration values was performed as described by [29]. The evaluated frequencies ranging from 30 Hz to 100 Hz showed that the acceleration increases with the square of frequency as expected as well as the mass of the inserted unbalances. Especially at 60 Hz, the turbine provides accurate vibrational waves in absence of higher harmonics. The frequency spectrum is broader at lower frequencies (40 Hz) and should be improved in future designs.

There are further ways of improving the actuation setup. The influence of (1) possible friction within the turbine itself, (2) a delay between the adjusted pressure of compressed air at the proportional pressure regulator and the turbine due to the long (> 5 m) pressure supply tubes, (3) a decrease in acceleration at natural frequencies of the turbine on the stability of the operating actuation frequency, (4) an improved feedback loop of the light sensor signal to the pressure regulator will be investigated in future studies.

The current turbine design allows a placement of the housing within the holes of a commercially available 4-channel flex coil (Siemens healthineers). Design alterations of the actuator should be considered for placement within the holes of a Body 18 coil. Modifications in the CAD are feasible, e.g. to decrease the outer dimensions of the housing or to develop a more flat design. The generated force could remain the same despite a decreased distance of the unbalance to the rotational center, as the mass of the unbalance could simultaneously be increased. Another solution is the application of intervention coils. The coil’s biopsy windows offer sufficient space for placement of the actuator. Future alterations of the turbine design might also explore the possibility of a multi frequency vibration. Another rotating mass filling two opposing quarters can be placed besides the existing unbalance within the turbine. Thus, a bi-frequency setup could be achieved.

Overall, our proposed pneumatic turbine generated vibration amplitudes sufficiently large to entirely penetrate the phantom during our feasibility studies for frequencies ranging from 50 Hz to 80 Hz at 3 T. As seen in the phase images, the inclusion is clearly distinguishable from the background material due to the larger wave length within the inclusion. A further evaluation of different actuation frequencies and reconstruction of elasticity maps remains future work. Further research could usefully explore an optimal ratio of the weight of unbalance to turbine size for sufficiently large but tolerable wave actuation with respect to clinical applications.

The second aim of the study was to determine, if silicone based materials can be used as MR-compatible tissue-mimicking material for MRE evaluation. The measured shear moduli of the silicone samples are in the range of human soft tissue values reported by literature MRE measurements and ex vivo bio-mechanical tests [42–46]. It is possible, therefore, to manufacture custom-built phantoms with known elasticities and arbitrary shapes. Since commercial phantoms tend to dehydrate over time [14], their elasticities may be influenced and given elasticity values can change, leaving room for uncertainties regarding the shear modulus. Here, the silicone-based custom-made phantoms offer an excellent alternative. The manufacturing process was kept simple and repeatable.

Additionally, silicone yields sufficient signal for MR imaging. It is possible to alter the phantom for multimodal imaging, e.g. by introducing scatter particles in the material for ultrasound (US) imaging. Future experimental work will determine the influence of scatter particles with regard to elasticity values. Hence, a correlation of MR and US elastography may be performed on such a multimodal phantom.

MRE is a unique technique for the identification of various pathologies, as viscoelastic characteristics may vary between healthy and diseased tissue. The quantification of the shear modulus is therefore promising as a further independent parameter for MR diagnostics in a variety of clinical applications. In conclusion, this work demonstrates the technical feasibility of a novel MRI-compatible set up based on centrifugal force for the quantification of spatial stiffness of soft tissues. We tested our design on an in–house developed anthropomorphic phantom that closely match elasticity values of human tissues. The actuator is easy to set up, does not interfere with the imaging procedure and can be integrated into existing clinic equipment. The design is adaptable and reproducible through low-cost 3D printing. It has also been aimed to meet clinical demands and can readily be used at a field strength of 3 T. However, our preliminary results need to be further validated in volunteer studies.
